# Aging Deteriorates Blood Brain Barrier Function and Polarizes Adaptive T Cell Expansion Contributing to Neurocognitive Damage in Experimental Cirrhosis

**DOI:** 10.14336/AD.2024.0932

**Published:** 2025-10-12

**Authors:** Sebastián Martínez-López, María Salud García-Gutiérrez, Francisco Navarrete, Isabel Gómez-Hurtado, Pedro Zapater, Enrique Ángel, Oriol Juanola, Juan L López-Cánovas, Paula Boix, Manel C Hadid, Amaya Puig-Kröger, Manuel D Gahete, Jorge Manzanares, Esther Caparrós, Rubén Francés

**Affiliations:** ^1^Grupo de Inmunobiología Hepática e Intestinal, Dpto. Medicina Clínica e Instituto IDIBE, Universidad Miguel Hernández, Alicante, Spain.; ^2^IIS ISABIAL, Hospital General Universitario de Alicante, Alicante, Spain.; ^3^Instituto de Neurociencias, Universidad Miguel Hernández-CSIC, Alicante, Spain.; ^4^CIBERehd, Instituto de Salud Carlos III, Madrid, Spain.; ^5^Department of Cell Biology, Physiology and Immunology, University of Córdoba, Córdoba, Spain.; ^6^Maimónides Institute of Biomedical Research of Córdoba (IMIBIC), Córdoba, Spain.; ^7^Reina Sofía University Hospital, Córdoba, Spain.; ^8^CIBER Pathophysiology of Obesity and Nutrition (CIBERobn), Córdoba, Spain.; ^9^Unidad de Inmunometabolismo e Inflamación, IIS Gregorio Marañón, Hospital Gral Univ Gregorio Marañón, Madrid, Spain.

**Keywords:** Aging, cirrhosis, liver-brain axis, neuroinflammation, behavior

## Abstract

Cirrhosis incidence is significantly increased with age and frequently complicated with neurocognitive dysfunction. We have evaluated the contribution of aging to neuroinflammation in the liver-brain axis in advanced chronic liver disease. Young (6-week-old) and old (9-month-old) mice were included in a 12-week protocol of CCl_4_-induced cirrhosis. Liver damage, neuromotor and cognitive capacities, blood brain barrier integrity and function, liver and brain T cell subpopulations and ammonia levels were evaluated. Timp1 and Acta2 gene expression was upregulated in old cirrhotic mice. Increased liver damage was confirmed histologically by Sirius red staining, expression of alpha-SMA, collagen 1-alpha1 and vimentin in aged CCl_4_-treated mice. Aging further compromised the neuromotor and cognition capabilities in cirrhotic animals. Stress axis components Crh and its receptor Nr3c1 gene expression levels were upregulated in the paraventricular nucleus and hippocampus of old cirrhotic mice. CCl_4_-damage significantly increased ammonia levels in the liver, brain and serum of cirrhotic mice. Circulating ammonia was significantly higher in old cirrhotic mice. Significant correlations were established between brain ammonia, neuromotor capabilities and results on the object recognition tests. A decreased integrity of blood brain barrier was accompanied by astrocyte activation and increased apoptosis-linked cleaved Caspase 3 in old cirrhotic mice. Liver resident CD4^+^ T-cell subpopulations were contracted in cirrhosis, although they showed a pro-inflammatory Th17 profile. Liver and brain resident CD8^+^ T-cell subpopulations were expanded in old cirrhotic animals, along with reduced tissue cytolytic activity. CD8^+^ T cell expansion and reduced perforin levels in the brain correlated with neuromotor and cognitive dysfunction. In conclusion, aging aggravates liver fibrosis, worsens neuromotor and cognitive functions and shifts liver and brain adaptive T cell profiles compromising the BBB integrity in experimental advanced chronic liver disease. Results strengthen the impact of aging in the liver-brain axis and neuroinflammation in cirrhosis.

## INTRODUCTION

Liver cirrhosis is the end-stage disease caused by continuous liver damage of different origin. Patients with cirrhosis are at risk of clinically relevant complications, which define the progression of the compensated disease towards a decompensated cirrhosis, worsening their prognosis [1, 2]. A mild neurocognitive dysfunction is frequent in patients with cirrhosis. This condition, named minimal or covert hepatic encephalopathy affects quality of life [3] and predicts the development of overt hepatic encephalopathy [4], defined by the dysfunction on different neuromotor and cognitive capacities leading to worse prognosis, quality of life and patient survival [5, 6].

The liver requires an efficient immune surveillance that allows maintaining a tolerogenic state against commensal antigens, while providing clearance of potential pathogenic burden [7, 8]. The gut microbiota shift shown in cirrhosis towards pathogenic clusters [9, 10] mediates hepatic antigen presenting cell (APC) transition to a persistent immune activation, modification of the hepatokine microenvironment profile, and the differentiation of pro-inflammatory T cell responses [11-14]. The compromised immune surveillance activity in the liver favors the exhaustion of immune cells functional capacity. On the other hand, the progressive hepatocyte necrosis induces a hyper-activation of the systemic inflammatory response and the negative regulation of anti-inflammatory pathways [15]. In turn, the systemic inflammatory response may have an impact on neural functions by altering blood-brain barrier (BBB) permeability [16], thus facilitating brain T cell recruitment and promoting neurologic disturbances in the context of advanced chronic liver disease.

Liver function is compromised during aging, as that of many other organs [17]. The incidence of advanced chronic liver disease is significantly increased with age, and it constitutes a factor of worse prognosis [18]. The liver aging process is modulated by genome and epigenome changes, which contribute to the disruption of mitochondrial function and nutrient sensing pathways, resulting in cellular senescence and low-grade inflammation [19]. Older cirrhotic rats display further worsening of the hepatic inflammatory phenotype, evidenced by a notable rise in the recruitment of proinflammatory macrophages and a reduction in the expression of pro-resolution cytokines within the same cell subset [20]. However, tissue resident T cell subsets have been less explored in this setting.

The present study aimed at characterizing the contribution of aging to the T-cell subset expansion in the liver-brain axis and to neurocognitive impairment in advanced chronic liver disease.

## MATERIALS AND METHODS

### Animals

Male C57Bl/6J (Harlan, Barcelona, Spain) were included in a 12-week study protocol and were grouped by age as young (6-week-old) or old (9-month-old). Mice were caged at a constant room temperature of 21°C and exposed to a 12:12 light/dark cycle. Adult mice weighing 20-22g were fed with standard rodent chow and treated with 0.25mmol/L phenobarbital in tap water along study protocol. After 4 weeks, animals were subjected to experimental cirrhosis induction with two weekly weight-controlled doses of CCl_4_ (Sigma Aldrich, Madrid, Spain) intragastrically, administered as described previously [21]. Control animals received mineral oil for that period. Animals were sacrificed when severely ill, and death was suspected to be imminent. After protocol completion animals underwent a set of behavioral tests to evaluate motor and cognitive impairment. Laparotomies were performed under anaesthesia with isoflurane at week 12 as described. During this process, blood samples were collected by cardiac puncture before perfusion and the brain was removed for molecular biology and histology experiments. After hepatic perfusion, livers were set aside either for molecular biology and histology experiments or flow cytometry experiments. Animals received care according to the criteria outlined in the Guide for the Care and Use of Laboratory Animals. The study was approved by the Animal Research Committee of Universidad Miguel Hernandez (Alicante, Spain) with approval number 2021/VSC/PEA/0239.

### Flow cytometry

Perfused livers were digested *in vivo* with collagenase A (Merck-Millipore, Burlington, MA, USA) as previously described [22]. Resultant digested livers were excised, and an in vitro digestion with the same buffer containing collagenase A was performed at 37°C for 30 min. The liver cell solution was then filtered by using 100 µM nylon strainers and collected in cold Kreb’s solution containing 25 mM HEPES. The cell suspension was centrifuged at 50x g for 5 min, and non-parenchymal cells were separated by collecting the supernatants and then centrifuged at 800x g for 10 min. Resultant pellets were resuspended in 10 mL of Percoll 40% and non-parenchymal cells were enriched and isolated by differential centrifugation at 800x g for 25 min. Cells from the pellet were collected, washed with phosphate-buffered saline without Ca^2+^ and Mg^2^ (PBS), and resuspended in PBS supplemented with 2% fetal (FBS) and 2 mM ethylenediaminetetraacetic acid (EDTA) for cytometric staining. Then, isolated cells were divided equally and incubated with the correspondent antibodies of each cytometry panel (Supplementary Table 1). To evaluate the T helper (Th) CD4 differentiation profile, cells were stimulated with phorbol myristate acetate/ionomycin (50 ng/mL and 1 μg/mL, respectively) (Sigma-Aldrich, Madrid, Spain) for 5h, and Golgi traffic-blocked with monensin (BD Golgi STOP, BD Biosciences). Then, cells were then fixed, permeabilized, and stained intracellularly. Samples were analyzed in the Omics facility of the Instituto de Neurociencias de Alicante in a FACSAria II flow cytometer operated by FACSDiva software (BD Biosciences, San Diego, CA, USA).

### Biochemical markers

Serum levels of alanine transferase (ALT), aspartate transferase (AST), urea and albumin (ALB) were determined in 200 µL of total blood using an automatic liquid biochemistry analyzer (Skyla Vb1, CVM practice, Navarra, Spain) following manufactures instructions.

### Ammonia levels

Remaining blood was quickly processed by centrifuging samples for 10 minutes at 1000x g at 4°C. Then, the serum was transferred to a new tube and flash frozen for later determinations. Liver or brain tissue were lysed in PBS containing cOmplete™ Protease Inhibitor Cocktail (Merck, Darmstadt, Germany). Tissue lysates or plasma were tested for ammonia levels using the Ammonia Assay Kit ab83360 (Abcam, Cambridge, UK), following the manufacturer’s instructions.

### ELISAs

Protein levels of matrix metallo-proteinase (MMP)-9 and MMP12 were quantified by the Quantikine Mouse Total MMP-9 kit (MMPT90, Biotechne) and the Mouse MMP-12 Elisa kit (ab213878, Abcam) respectively according to the manufacturer's instructions. Liver and brain lysates were obtained as described before and diluted 5-fold and 2-fold respectively. The remaining serum from ammonia determination was diluted 20-fold for the determination of the analytes. Plates were analyzed in an EPOCH2 microplate reader (BioTek). Each sample was analyzed in duplicates.

### Histological analysis

Sirius Red staining was performed using a Picro Sirus Red Stain Kit (ab150681) from Abcam in 5-µm sections of paraffin-embedded mouse liver according to the manufacturer’s instruction. In brief, after deparaffinization sections were incubated in Picro Sirus Red solution for 60-min and followed by two times quickly rinsing in acetic acid solution and absolute alcohol.

Immunohistochemical (IHC) and immune-fluorescence (IF) assays were carried out in 5-µm sections of paraffin-embedded mouse liver or brain tissue. The slides were incubated with primary antibodies for key proteins involved in hepatic or brain homeostasis and disease. As secondary antibodies, sections were incubated with the correspondent biotinylated antibodies (Palex Medical SA, Sant Cugat del Vallés, Spain) for IHC or with Alexa-488 or Alexa-594 modified antibodies for IF. Dilutions and antibody references can be followed in Supplementary Table 2. IHC slides were incubated with avidin-biotin-HRP complex ABC kit (Vector Laboratories Inc., Burlingame, CA) and developed with peroxidase substrate 3,3′-diaminobenzidine (Vector Laboratories Inc.). Nuclei were stained by incubating the sections in Harris hematoxylin (Leica Biosystems Richmond Inc., Richmond, IL) or DAPI (Thermo Fisher Scientific) respectively. Staining without primary antibody was used as negative control.

Images were obtained in a camera-assisted optic Leica DMR microscope (Leica Biosystems, Richmond Inc.). A semi-quantitative analysis of protein expression was performed using the ImageJ software (https://rsbweb.nih.gov).

### Neuromotor and cognitive evaluation

#### Open field (OF)

This test was used to evaluate general motor activity. Mice were individually placed at the center of methacrylate square boxes with white Plexiglas floor (25 x 25 x 25 cm) and their behavior was videotaped for 15 min. The total distance travelled by the mice was monitored and analyzed directly with the SMART (Spontaneous Motor Activity Recording and Tracking) v2.5.3 software system (Panlab, Barcelona, Spain).

#### Neurological severity score (NSS)

The motor items of the neurological severity score (NSS) were used in the mice to evaluate the severity of their neurological damage and its consequences on motor coordination, as previously described [23] and shown in Supplementary Table 3. A higher score corresponds to a more severe neurological injury.

#### Elevated plus-maze test (EPM)

This test was used to evaluate anxiety-like behavior. A black Plexiglas apparatus consisting of 4 arms (29 cm long x 5 cm wide), 2 open and 2 closed, set in a cross from a neutral central square (5 × 5 cm) elevated 40 cm above the floor was used. Light intensity in the open and closed arms was 45 and 5 lx, respectively. Mice were placed in the central square facing one of the closed arms and tested for 5 min. The time spent in the open arms of the maze (as percentages of total test time) was determined as a measure of anxiety-like behavior, whereas the total entries in the open and closed arms were considered an indirect measure of locomotor activity, as previously reported [24].

#### Novel Object Recognition (NOR)

During the habituation phase, mice were exposed to a square open field cage (50 x 50 x 50 cm) with two identical objects in texture, color, size, and shape (named familiar objects), and the exploration time of each object was recorded for 5 min. After 24 h, the mice were re-exposed to the same cage, switching one of the familiar objects with a new one. Similarly, the exploration time of both objects was measured for 5 min. The period during which the animal orientates the nose, sniffs, or touches the object with its front legs at a less or equal distance to 1 cm was defined as the exploration time. The discrimination index (DI) was calculated as the difference between time spent exploring the novel and familiar object divided by the total exploration time of the two objects according to the following formula: DI = (New object exploration time - Familiar Object exploration time) / (New object exploration time + Familiar Object exploration time). Discrimination ratio values ranged from -1 to 1. A score closer to -1 indicated a preference for the familiar object, while a score closer to 1 indicated the preference for the novel object. Thus, a high discrimination index reflects better memory retention for the familiar object.

### Quantitative PCR analysis

Total RNA was extracted using RNeasy Mini Kit (QIAgen) from cell pellets or liver samples. Quantitative PCRs were performed to evaluate the expression of *LSECtin/Clec4g*, as well as the key genes involved in hepatic disease. The reactions were performed in a 12.5-µL PCR mixture using qScript One-Step SYBR Green RT-qPCR (Quanta BioScience, Gaithesburg, Maryland) in a CFX Connect (Bio-Rad, Hercules, CA). β2-microglobulin was used as a housekeeping gene in all gene expression analyses. Relative expression was calculated with the 2^-ΔΔCt^ method. Primer pairs used in the study can be followed in Supplementary Table 4.

Relative gene expression of corticotropin releasing hormone (*Crh*) in the paraventricular nucleus (PVN), and glucocorticoid receptor (*Nr3c1*), brain-derived neurotrophic factor (*Bdnf*) and tropomyosin receptor kinase B (*Ntrk2*) in the hippocampus (HIP) were analysed. During laparotomies brains were removed from the skull and frozen over dry ice. Coronal sections (500 μm) containing the regions of interest were cut in a cryostat (-10 °C) according to Paxinos and Franklin atlas (Franklin and Paxinos, 2001), mounted onto slides, and stored at -80 °C. Sections were microdissected to obtain the PVN and the HIP following the method described by Palkovits (Palkovits, 1983). Total RNA was obtained from brain micropunches using TRI extraction reagent (Applied Biosystems, Madrid, Spain). Reverse transcription to complementary DNA (cDNA) was carried out with the High-Capacity cDNA Reverse Transcription Kit following the manufacturer's instructions (Applied Biosystems). The relative abundances of the genes of interest were quantified in a StepOne Plus Sequence Detector System (Life Technologies, Madrid, Spain) using TaqMan probes (Supplementary Table 5). The reference genes used were 18S rRNA. As before, data for each target gene was normalized to the endogenous reference, and the fold change in target gene expression was determined using the 2^-ΔΔCt^ method.

### Western Blot

Liver and brain homogenates from mice samples were lysed with radioimmunoprecipitation assay buffer containing cOmplete™ Protease Inhibitor Cocktail (Merck, Darmstadt, Germany). Bradford protein assay (EMD Millipore Corp., Billerica, MA) was used to calculate protein concentration. Thirty micrograms of protein extracts were resolved under reducing conditions on 6% to 15%, sodium dodecyl sulfate-polyacrylamide gels depending on the size of the protein of interest. Then, gels were transferred to Immobilon-P membranes (Merck, Darmstadt, Germany). After blocking, membranes were incubated with primary antibodies overnight. β-actin (Sigma-Aldrich) was used as control. Membranes were incubated with horseradish peroxidase (HRP)-conjugated secondary antibodies (Cell Signaling Technology, Leiden, Netherlands). Membrane-attached peroxidase activity was detected by Immobilon Western Chemilum HRP Substrate (EMD Millipore Corp.). All images were obtained in a ChemiDOC XRS+ run under Image Lab software (Bio-Rad). Protein bands were quantified by densitometry using ImageJ (https://rsbweb.nih.gov). Band densities were expressed relative to total β-Actin protein. Dilutions and antibody references can be followed in Supplementary Table 6.

### Statistical analysis

Categorical variables are represented as frequency or percentages and continuous variables as mean ± standard deviation. The normality of continuous variables was evaluated using the Kolmogorov-Smirnov test (p<0.05). As the normal distribution was not confirmed, differences between groups were analyzed using the non-parametric Mann-Whitney U test. Multiple comparisons were analyzed according to Bonferroni correction. All reported p-values were two-sided, and p-values lower than 0.05 were considered to indicate significance. GraphPad Prism version 8.0 (San Diego, CA) was used for statistical analysis and graph design.

**Figure 1.**
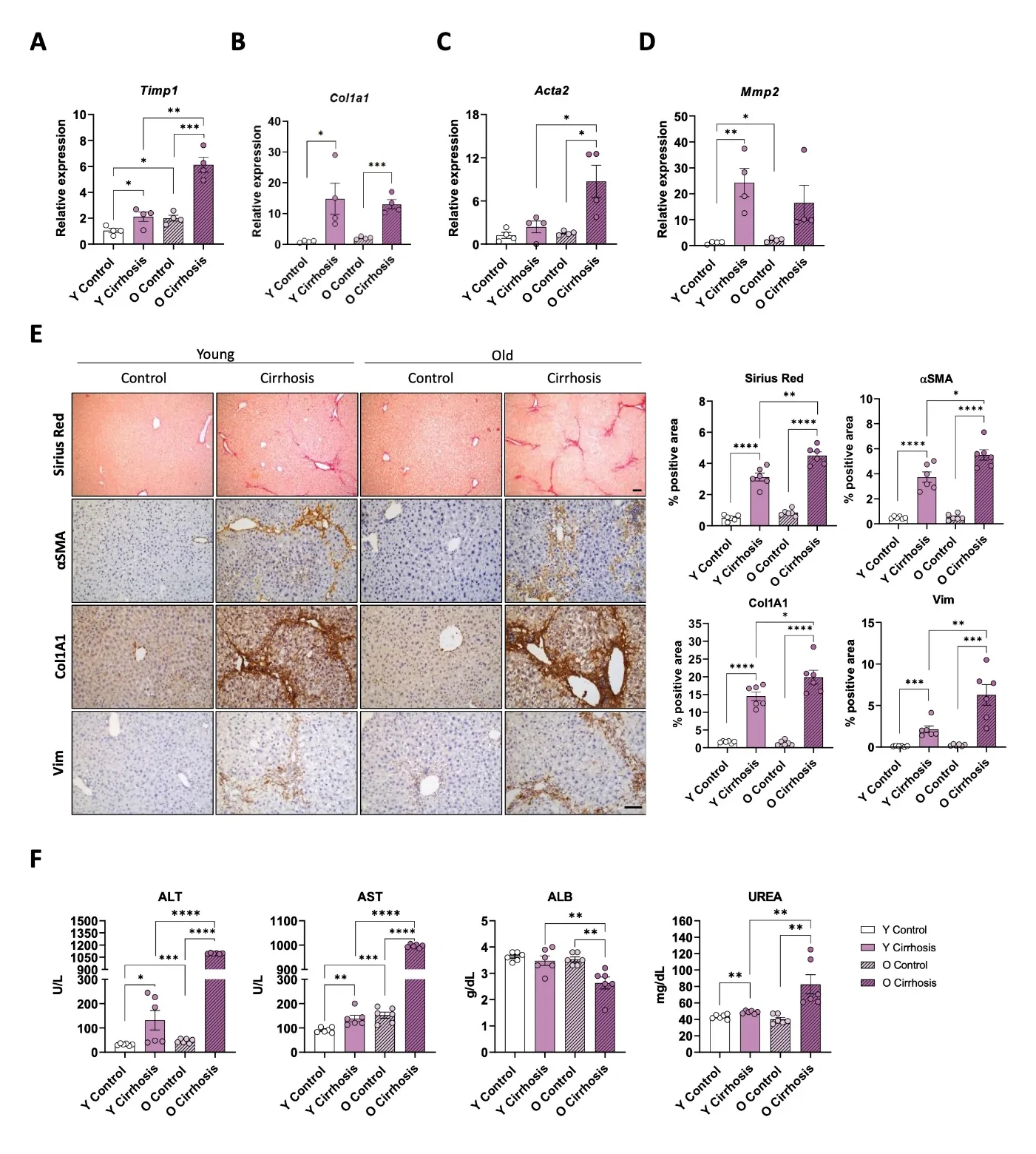
**Liver damage in young and old mice with cirrhosis**. (**A-D**) mRNA relative expression of *Timp1, Col1a1, Acta2* and *Mmp2* in total liver homogenates. Mean ± standard deviations are represented (4 animals/group). (**E**) Fibrosis-related stains in the liver. Representative liver images stained with Sirius red, α-SMA, collagen I and Vimentin and their respective quantifications. Signal was blindly measured in user-specified regions of interest (ROIs) as brown or red area percentage in respect to the total area of the ROI using the ImageJ software. Mean ± standard deviations are represented (6 animals/group). Scale bar = 100 µm. (**F**) Biochemical characterization liver function parameters in blood samples. Mean ± standard deviations are represented (6 animals/group). Abbreviations: Alanine transferase (ALT), Aspartate transferase (AST), albumin (ALB), Tissue inhibitor of metalloproteinases 1 (Timp1), Collagen 1 (Col1a1), Alpha smooth muscle actin (α-SMA and Acta2), Matrix metalloproteinase-2 (Mmp2), Vimentin (Vim), Young (Y) and Old (O). P values are indicated as follows (*) *p* < 0.05; (**) *p* < 0.01; (***) *p* < 0.001; and (****) *p* < 0.0001.

## RESULTS

### Aging aggravates liver fibrosis and function in ACLD

Liver damage was evidenced by CCl_4_ treatment by significantly increased expression levels of profibrogenic genes compared to control mice. Age also contributed to liver damage as a further upregulation of Timp1 and Acta2 in the liver of old cirrhotic mice compared to young ones (Fig. 1A-1D). Hepatic fibrotic damage was confirmed histologically by Sirius red staining, significantly increased expression of alpha-SMA, collagen 1-alpha1 and vimentin in CCl_4_-treated mice (Fig. 1E). As shown for profibrogenic genes, aged mice also evidenced a significantly worse liver injury compared to young cirrhotic animals. The assessment of liver function also revealed that the CCl_4_-induced altered hepatic enzymes levels were even greater in old cirrhotic mice (Fig. 1F).

**Figure 2.**
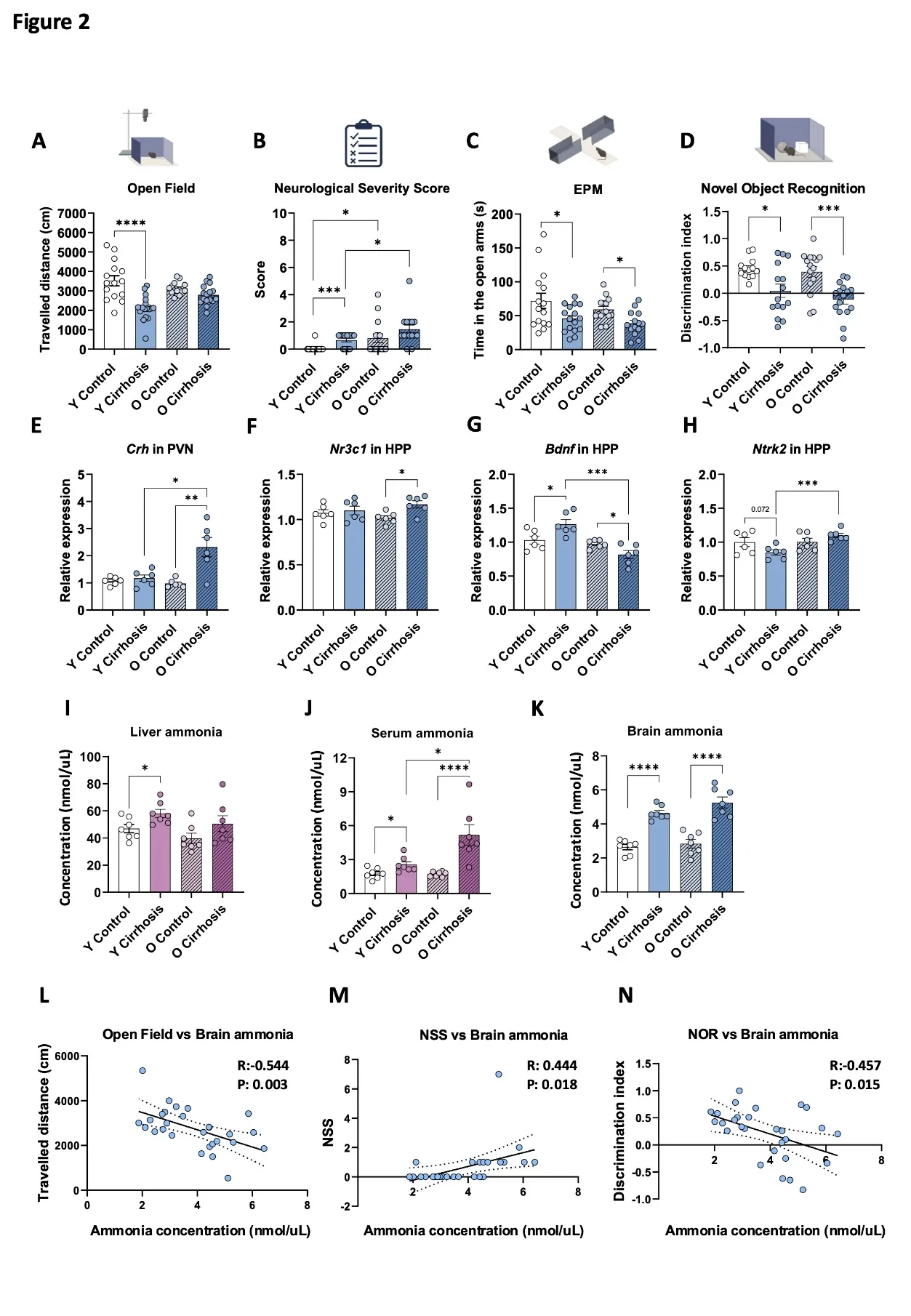
**Behavior alteration in young and old mice with cirrhosis**. (**A-D**) Panel of behavioral tests designed to evaluate alterations derived from liver disease and aging. The Open Field (A) and the Neurological Severity Score (B) evaluated motor performance and neurological state, respectively. The Elevated Plus Maze (C) evaluated anxiety-like behavior. The Object Recognition test (D) evaluated recognition memory. Mean ± standard deviations are represented (15 animals/group). (**E-H**) mRNA relative expression of *Crh, Nr3c1, Bdnf* and *Ntrk2* in either the PVN or the HPP of mouse brains. Mean ± standard deviations are represented (6 animals/group). (**I-K**) Ammonia levels in the liver, serum and brain respectively of the mice subjected to the behavioral tests. Mean ± standard deviations are represented (7 animals/group). (**L-N**) Correlation between brain ammonia levels and Open Field Test (L), Neurological Severity Score (M) and Object Recognition test (N) respectively. Abbreviations: Elevated Plus Maze (EPM), Corticotropin-releasing factor (Crh), Nuclear Receptor Subfamily 3 Group C Member 1 (Nr3c1), Brain-Derived Neurotrophic Factor (Bdnf), Neurotrophic receptor Tyrosine Kinase 2 (Ntrk2), Paraventricular nucleus (PVN), Hippocampus (HPP), Neurological Severity Score (NSS), Young (Y) and Old (O). P values are indicated as follows (*) p < 0.05; (**) p < 0.01; (***) p < 0.001; and (****) p < 0.0001.

### Neuromotor and cognitive capabilities worsen during aging in cirrhosis

Cirrhotic animals show significantly impaired neuromotor capabilities compared to control mice, as shown by a reduced travelled distance and an increased neurological severity score in the open field and motor evaluation tests, respectively (Fig. 2A-2B). Cirrhosis is also associated with significantly increased anxiety, as demonstrated by the lower time spent in the open arms of the elevated plus maze (Fig. 2C), and impaired cognitive function as revealed by a decreased discrimination index in the object recognition test (Fig. 2D). Aging further compromises the neuromotor capabilities of cirrhotic animals (Fig. 2A-2B). Anxiety and the cognition derangement induced by cirrhosis were also higher in old *vs* young cirrhotic animals, although differences did not reach statistical significance (Fig. 2C-2D).

Stress axis was significantly altered in old cirrhotic mice, as revealed by the increased Crh and its receptor Nr3c1 gene expression levels in the PVN and HIP, respectively, of these animals compared to old control mice and young cirrhotic animals (Fig. 2E-2F). Also of interest, Bdnf, involved in neuronal differentiation and synaptic connections (neuroplasticity), shows significantly decreased gene expression levels in the HIP of old cirrhotic mice compared to old control mice and young cirrhotic animals (Fig. 2G-2H). Notably, Bdnf increased in the HIP of young cirrhotic animals compared to young control mice (Fig. 2G). Regarding NtrK2, a tendency to decrease in old cirrhotic mice is observed compared to old control mice (Fig. 2H).

We next evaluated liver, brain and systemic ammonia levels, as metabolic factors associated with neurocognitive damage in cirrhosis. Induced liver damage significantly increased ammonia levels in the hepatic tissue of cirrhotic mice compared to control animals (Fig. 2I). Hepatic gene expression levels of several intermediaries of urea cycle were evaluated and are represented in Supplementary Figure 1. Old cirrhotic mice showed significantly reduced gene expression levels of all evaluated substrates. Cirrhosis was also associated with increased serum (Fig. 2J) and brain (Fig. 2K) ammonia levels compared to control animals. Only circulating levels of ammonia were significantly higher in old *vs* young cirrhotic mice, without differences in liver and brain ammonia levels according to age. Considering all groups of mice, significant correlations could be established between brain ammonia levels and the neuromotor capabilities of mice (Fig. 2L-2M) and their results on the NOR test (Fig. 2N).

As ammonia producing microbiota contribute to hyperammonemia in cirrhosis, we evaluated gut barrier integrity in young *vs* old cirrhotic mice. Cirrhosis induced a significant downregulation of tight-junction proteins Claudin 1 and ZO-1 and the upregulation of the gut vascular marker PV1 compared to control animals. Aging didn’t show a significantly worsening effect on these proteins, although the leukocyte infiltration was significantly higher in aged *vs* young cirrhotic mice. In this line, serum LPS levels were increased in aged cirrhotic animals, suggesting an increased gut permeability in cirrhosis during aging (Supplementary Fig. 2).

### Blood brain barrier integrity is compromised in experimental ACLD

**Figure 3.**
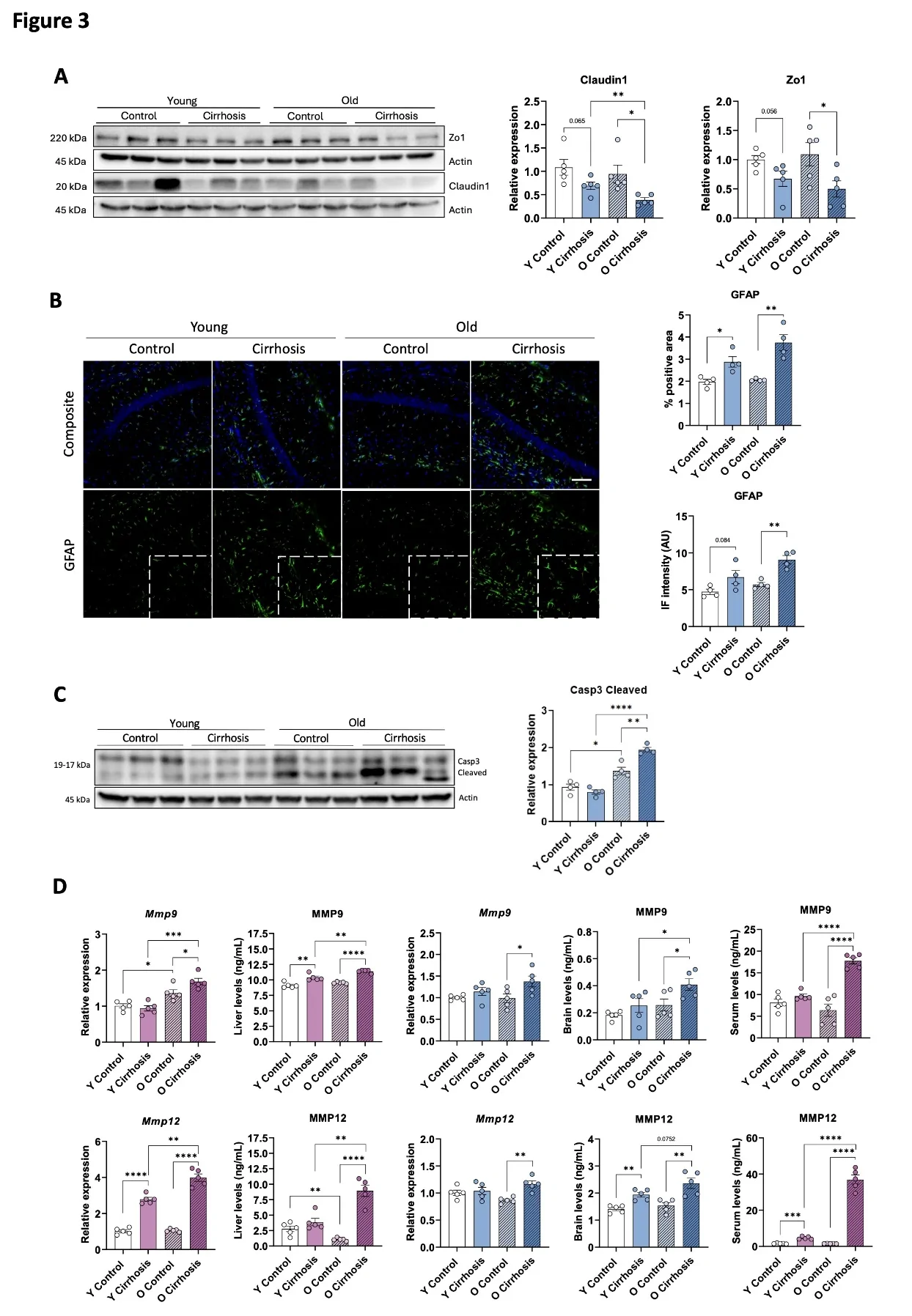
**Blood brain barrier alterations in young and old mice with cirrhosis**. (**A**) Representative Western blot of three biological replicates and protein relative expression of Zo1 and Claudin1 in brain tissue homogenates. Band densitometry is shown by the image as relative to b-actin levels. Mean ± standard deviations are represented (5 animals/group). (**B**) Representative images of hippocampus stained with GFAP and its respective quantification. Signal was blindly measured in user-specified ROIs as green area percentage in respect to the total area of the ROI using the ImageJ software. Nuclei, stained by DAPI, are represented in blue. Mean ± standard deviations are represented (4 animals/group). Scale bar = 100 µm. (**C**) Representative Western blot of three biological replicates and protein relative expression of Cleaved Casp3 in brain tissue homogenates. Band densitometry is shown by the image as relative to b-actin levels. Mean ± standard deviations are represented (4 animals/group). (**D**) Gene and protein expression levels of MMP9 and MMP12 in liver and brain, and serum of all study groups. Mean ± standard deviations are represented (5 animals/group). Abbreviations: Zonula occludens 1 (Zo1), Glial fibrillary acidic protein (GFAP), Immunofluorescence (IF), Caspase 3 (Casp3), matrix metallo-proteinase (MMP), Young (Y) and Old (O). P values are indicated as follows (*) p < 0.05; (**) p < 0.01; (***) p < 0.001; and (****) p < 0.0001.

Given the increased ammonia levels in brain tissue, we evaluated the BBB integrity in cirrhotic mice. The expression of tight-junction proteins Zo1 and Claudin1 were significantly downregulated in brain’s prefrontal cortex homogenates of cirrhotic mice. Aging, in turn, contributed by further downregulating the expression of these proteins in cirrhotic mice (Fig. 3A). Accordingly, an increased activation of astrocytes was observed by GFAP expression in cirrhotic brain tissue, with a tendency to a higher increment in aged cirrhotic mice (Fig. 3B). In line with these results, the apoptosis biomarker cleaved Casp3 is significantly increased in old cirrhotic mice (Fig. 3C).

To provide a potential link between liver damage and BBB increased permeability, we measured liver, serum and brain levels of MMP-9 and MMP-12, which have been implicated in BBB deterioration and aging-associated neuroinflammation. Whereas MMP9 was slightly upregulated in the liver, serum and brain of young cirrhotic *vs* control mice, its gene and protein expression levels in aged cirrhotic mice were significantly increased compared to aged control mice and to young cirrhotic mice (Fig. 3D). Similarly, MMP12 gene and protein expression levels in liver, brain and serum of aged cirrhotic mice were significantly and consistently increased compared to young cirrhotic mice (Fig. 3D).

### Aging shifts liver and brain adaptive T cell profiles in ACLD

**Figure 4.**
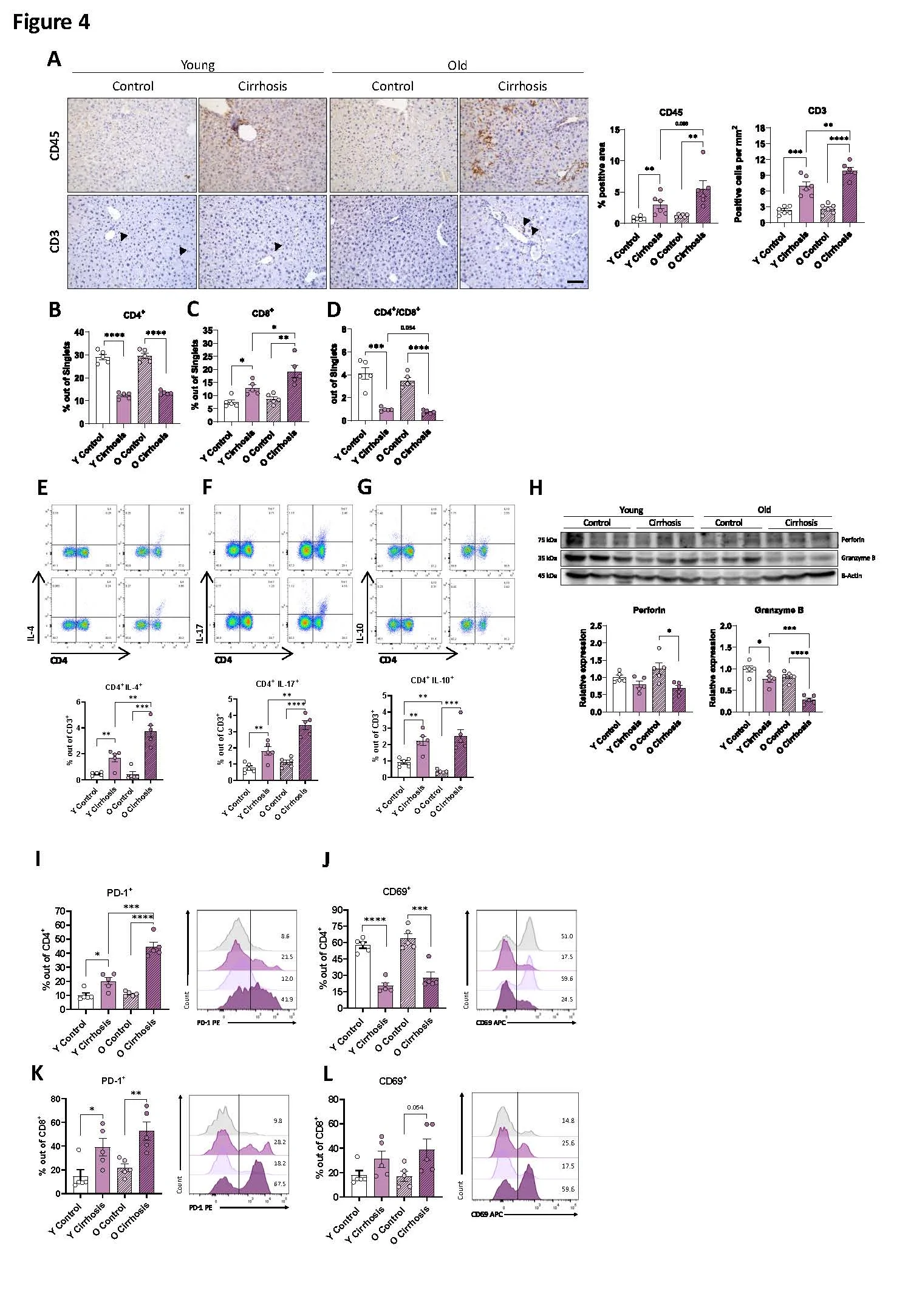
**Liver immune alterations in young and old mice with cirrhosis**. (**A**) Representative liver images stained with CD3 and CD45 and their respective quantifications. Signal was blindly measured in user-specified ROIs as brown area percentage in respect to the total area of the ROI using the ImageJ software. Mean ± standard deviations are represented (6 animals/group). Scale bar = 100 µm. (**B-C**) Flow cytometry analysis of T CD4^+^ (B) and CD8^+^ (C) cells from liver tissue. Mean ± standard deviations are represented (5 animals/group). (**D**) Ratio between CD4^+^ and CD8^+^ cells from liver tissue. (**E-F**) Representative dot plot images from flow cytometry analysis of T CD4^+^ cells expressing either IFNγ^+^ (E), IL-17^+^ (F) or IL-10^+^ (G). Data is expressed as percentage of positive cells of the previous gate. Mean ± standard deviations are represented (5 animals/group). (**H**) Representative protein relative expression of Perforin and Granzyme B by Western blot in liver tissue homogenates. Band densitometry is shown by the image as relative to b-actin levels. Mean ± standard deviations are represented (5 animals/group). (**I-L**) Representative histogram images from flow cytometry analysis of T CD4^+^ expressing PD-1^+^ (I) or CD69^+^ (J) and CD8^+^ expressing PD-1^+^ (K) or CD69^+^ (L). Mean ± standard deviations are represented (5 animals/group). Abbreviations: Cluster of differentiation (CD), Interferon Gamma (IFNγ), Interleukin (IL), Programmed cell death protein 1 (PD-1) Young (Y) and Old (O). P values are indicated as follows (*) p < 0.05; (**) p < 0.01; (***) p < 0.001; and (****) p < 0.0001.

Cirrhosis induces the increase of the T cell compartment in the liver compared to control animals (Fig. 4A). Aging contributes by further expanding the CD3^+^ cell population, as shown by the histologic analysis of liver sections from young and old cirrhotic mice. We characterized this CD3^+^ T cell subpopulations in the liver of cirrhotic mice. The CD4^+^ T cell population was contracted in cirrhosis, regardless of mice age (Fig. 4B). On the contrary, the CD8^+^ T cell subpopulation was significantly expanded in cirrhotic mice and age contributed to further increase this percentage (Fig. 4C). The ratio CD4^+^/CD8^+^ T cells in the liver of cirrhotic mice was significantly downregulated during cirrhosis (Fig. 4D). In parallel, innate monocyte and neutrophil subpopulations were reduced in the liver and brain of aged *vs* young healthy mice, whereas aging during cirrhosis induced an increase of monocyte and neutrophil hepatic infiltration (Supplementary Fig. 3),

Though we observed a contraction in the hepatic CD4^+^ T cells in cirrhotic *vs* control animals, intracellular expression of cytokines determining T CD4^+^ cells subpopulations showed an increase in Th2 and Th17 phenotypes in cirrhotic mice (Fig. 4E-4F). The tolerogenic T cell subset was also differentially increased (Fig. 4G). Aging induced a significant further increment in pro-inflammatory immunophenotypes whereas aged cirrhotic mice failed in expanding the counterbalancing tolerogenic IL-10-producing T cells. Regarding the cytotoxic activity, granzyme and perforin showed a reduction in cirrhosis compared to controls that was more drastic in aged mice (Fig. 4H). We also characterized the expression of cell surface markers for T cell activation state (Fig. 4I-4L). While PD1 expression in CD4^+^ and CD8^+^ T cells was upregulated in cirrhosis, the percentage of cells expressing CD69 was significantly reduced in CD4^+^ T cells. CD69 expression was significantly increased in CD8^+^ T cells in aged cirrhotic animals.

**Figure 5.**
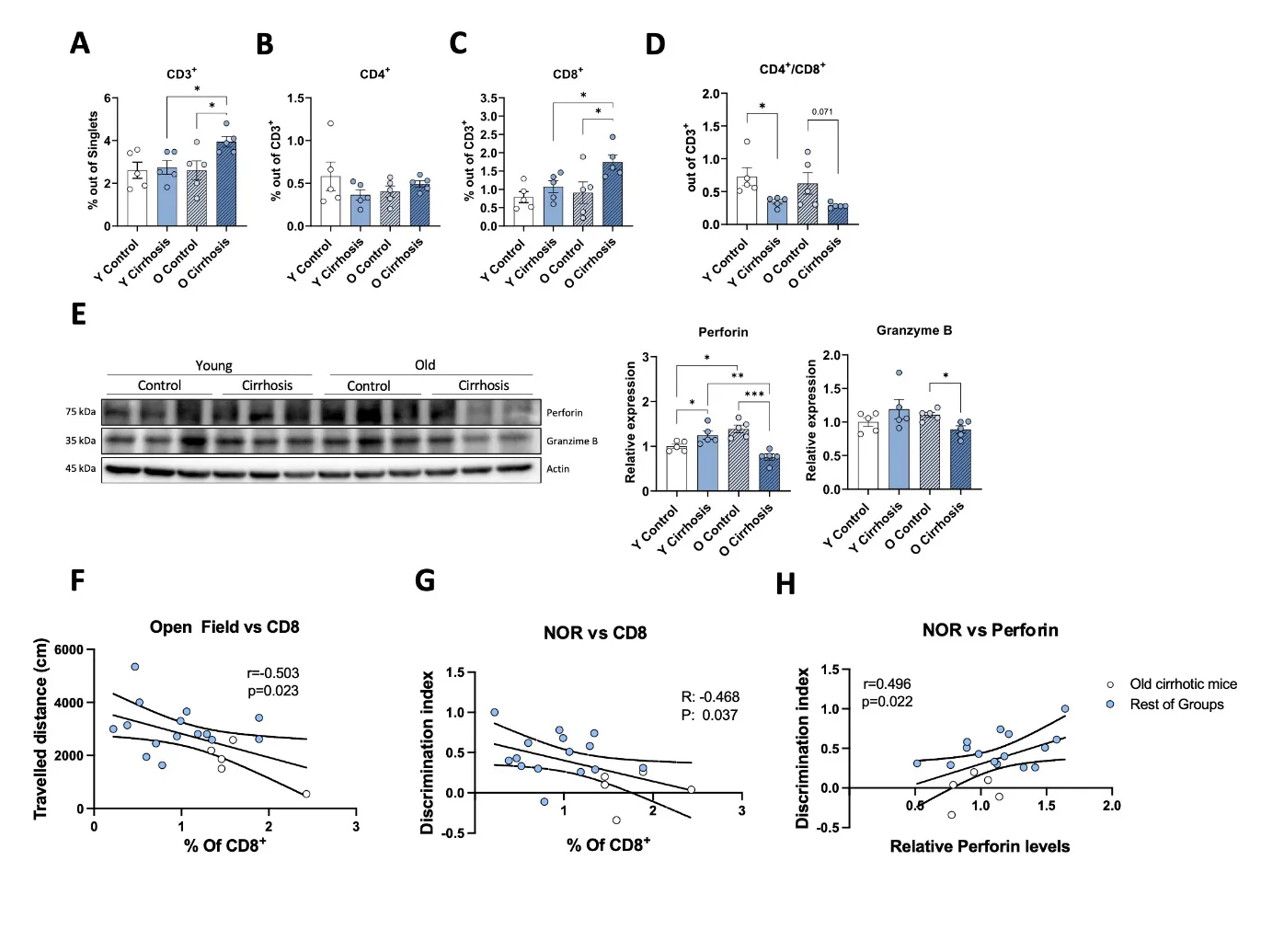
**Brain immune alterations in young and old mice with cirrhosis**. (**A-C**) Flow cytometry analysis of T CD3^+^ (A), CD4^+^ (B) and CD8^+^ (C) cells from brain tissue. Mean ± standard deviations are represented (5 animals/group). (**D**) Ratio between CD4^+^ and CD8^+^ T cells from brain tissue. (**E**) Representative protein relative expression of Perforin and Granzyme B by Western blot in brain tissue homogenates. Band densitometry is shown by the image as relative to b-actin levels. Mean ± standard deviations are represented (5 animals/group). (**F**) Correlation between percentage of CD8+ T cell population in brain and results in the Open Field test for all included animals. (**G**) Correlation between percentage of CD8+ T cell population in brain and results in the Object Recognition test for all included animals. (**H**) Correlation between perforin levels in the brain and results in the Object Recognition test for all included animals. Old cirrhotic mice are represented in white dots whereas the rest of groups are represented in colored dots. Abbreviations: Cluster of differentiation (CD), Young (Y) and Old (O). P values are indicated as follows (*) p < 0.05; (**) p < 0.01; (***) p < 0.001; and (****) p < 0.0001.

The expansion of CD3^+^ T cells in the brain of cirrhotic animals is only observed in aged mice, without significant differences between the rest of groups (Fig. 5A). This increment was due to the CD8^+^ T cell subset, as no differences were found between groups regarding the percentage of CD4+ T cell population (Fig. 5B-5C). As in the liver, the ratio CD4^+^/CD8^+^ T cells in the brain of cirrhotic mice was significantly downregulated during cirrhosis (Fig. 5D). Evaluation of effector cytotoxic activity mediated by perforin and granzyme showed a differential pattern in young and old cirrhotic mice compared to their controls. While young cirrhotic mice significantly increased perforin in response to liver damage, old cirrhotic mice showed a significantly decreased expression of perforin and granzyme in brain tissue (Fig. 5E).

The percentage of CD3^+^CD8^+^ T cell population in the brain showed an inverse correlation with the results obtained in the open field and object recognition tests performed by all included animals. Mice with increased numbers of CD8^+^ T cells showed reduced motor capabilities, as evidenced by reduced travelled distances (Fig. 5F), as well as memory capacities, obtaining worse scores in the discrimination index (Fig. 5G). Similarly, reduced levels of perforin in the brain, as observed in old cirrhotic mice, inversely correlated with object recognition capacities (Fig. 5H).

To support the CD8^+^ T cell expansion in aged cirrhosis we evaluated the concentration of CD8^+^ T cells recruiting chemokines CCL3 and CXCL10. Like the CD8^+^ T cell subpopulation, both chemokines were significantly upregulated in liver and brain of old vs young cirrhotic animals (Supplementary Fig. 4).

## DISCUSSION

In the present study, we have explored the effects of aging on liver damage, neuromotor and cognitive performance, as well as hepatic and brain T cell functionality during experimental cirrhosis. In addition to an altered hepatic ammonia metabolism, the proinflammatory phenotype of CD4^+^ T cells and the expansion of a CD8^+^ T cell subpopulation with reduced cytolytic activity in the liver is accompanied with a more deteriorated BBB function and the recruitment of dysfunctional CD8^+^ T cells in the brain of old compared to young cirrhotic mice. This polarized cellular adaptive immune infiltration may favor an increased neuromotor and cognitive stress during aging in cirrhosis.

Liver cirrhosis is considered as the end-stage of chronic liver disease of distinct etiology developed over the years [25]. Therefore, its incidence is significantly increased with age and, along with an age-derived diminished liver function, the number of disease complicating events augments in parallel [18]. Among these complications, neuromotor and cognitive damage, referred to as hepatic encephalopathy, is frequently developed in cirrhotic patients [26]. Thus, we have aimed at exploring the contribution of aging to the imbalance of the liver-brain axis and related pathophysiological events occurring during cirrhosis.

Fibrotic damage in aged mice exposed to CCl_4_-induced injury is enhanced compared to young cirrhotic mice. Previous studies have shown that the reduced expression of Sirtuin I on hepatocytes and hepatic stellate cells (HSCs) exacerbates fibrosis in aged mice with alcoholic liver disease [27], or that aggravated portal hypertension and the increase in the intrahepatic vascular resistance in aged mice are associated with expanded deposition of extracellular matrix in a more advanced disease model [20]. In line with this, while no differences are observed in matrix metalloproteinase (Mmp)-2 gene expression levels between young and old cirrhotic animals in our study, the expression of Timp1 is much higher in old cirrhotic mice suggesting a more decompensated balance between matrix degradation and the inhibition of such proteolytic activity in aged cirrhosis [28, 29].

Although hepatic immune status has also been explored in aging during liver damage, main efforts have been directed towards the innate response [20, 30, 31], as responsible for the early interaction with the dysbiotic gut-derived microbial products that commonly reach the cirrhotic liver [32]. In this regard, it has been speculated that macrophages and macrophage-derived cytokines might be involved in initial liver fibrogenesis in chronically injured old mice [30]. However, the T cell compartment and its functional activity in the liver of aged cirrhotic mice had not been characterized so far. The expansion of CD3^+^ T cells observed in aged cirrhotic livers was mainly due to the hepatic CD8+ T cell subpopulation. Nevertheless, the CD4^+^ T cell subset showed a skewed balance towards IL-4 and IL-17-producing cells that, in contrast to young cirrhotic mice, old cirrhotic animals couldn’t counterbalance with the expansion of tolerogenic IL-10-producing CD4^+^ T cell subsets. This fact can contribute as well to the increased fibrotic damage observed in old mice exposed to CCl_4_ toxicity, since IL-17 activates hepatic profibrotic response by triggering JUN amino terminal kinase (JNK) [33, 34] and activates the STAT3 signaling pathway in HSCs to produce type 1 collagen [35], whereas IL-4 producing Th2 subsets have shown cytokine-mediated stimulation of TGF-β and the upregulation of MMP-9 in the liver [36, 37]. As mentioned, the resident CD8^+^ T cell subset was clearly increased in cirrhosis and further differentiated in old cirrhotic animals. This fact is supported by the significant increase of specific CD8^+^ T cell recruiting chemokines CCL3 and CXCL10 [38, 39], as observed both in the liver and brain of aged cirrhotic mice included in our study. While T cell activation and differentiation of proinflammatory phenotypes can be followed by PD1 and intermediate CD69 expression in liver Th cells [40] (which may correspond to the hepatic proinflammatory Th subsets observed in our study), their functional exhaustion has been associated with lower CD69 expression [41]. On the contrary, the CD69^+^ subpopulation within the T cytotoxic (Tc) cell compartment has been associated with a reduced cytolytic capacity due to chronic activation [42]. In agreement, T cell exhaustion as well as dysfunctional CD8^+^ T cells have been described in cirrhosis [43-45]. In our study, the increased percentage of exhausted CD4^+^ T cells and of dysfunctional CD8^+^ T cells, along with the attenuated perforin and granzyme B hepatic cytotoxic activity, identifies a differential immunophenotype of resident T cells in the liver of old cirrhotic mice and suggests a contributing role of an aging-specific hepatic T cell milieu polarization to the increased liver damage.

The liver-brain axis serves as connecting route for cirrhosis-associated neuromotor and cognitive damage [46]. From a clinical perspective, it has been shown that minimal hepatic encephalopathy, along with age and MELD score, are independently associated with impaired survival in patients with cirrhosis [47]. Also, blood-cerebrospinal fluid barrier presents a modified gravity in cirrhotic patients, favoring increased permeability and suggesting a mechanism for developing neurocognitive dysfunction [48]. Related to this, falls are associated with poor health-related quality of life, death, and disability in patients with cirrhosis, being hepatic encephalopathy a primary risk factor [49, 50]. In our study, aged cirrhotic mice showed more anxiety-like behaviour, neuromotor dysfunction and worse recognition of new objects than young cirrhotic animals. Precipitating factors such as hyperammonemia and a dysregulated hepatic urea cycle [51] were also affected by aging in our cirrhotic animals. In fact, cirrhotic mice show a decreased gut barrier integrity and, during aging, an increased gut leukocyte infiltration and permeability to substrates such as LPS. Higher brain ammonia levels correlated with neurocognitive and behavioral tests. In line with this, old cirrhotic mice showed reduced gene expression levels of stress and neuroplasticity markers. Crh has been reported to increase in hyperammonemic rats contributing to impair circadian rhythm and motor activity [52]. Similarly, the Bdnf/TrkB pathway has been involved in neuroinflammation enhancing the GABAergic neurotransmission in the cerebellum affecting motor function [53], whereas Bdnf/TrkB pathway restoration improves spatial learning and memory [54]. In fact, transmembrane tight-junction proteins (TJPs) in the BBB were dysregulated in aged cirrhosis. It has been shown that BBB permeability is increased in parallel to the neurologic damage not only in acute liver failure [55, 56], but also in chronic liver disease by induced systemic inflammation [16], providing evidence for brain T cell recruitment in cirrhosis [57]. A role in BBB deterioration and aging-associated neuroinflammation for MMP-9 and MMP-12 has also been given to MMPs [58, 59]. Our results showing that hepatic MMP-9 and MMP-12 concentration correlate with peripheral and brain levels suggest a potential route for liver damage induction of an altered BBB permeability and neuroinflammation, especially during aging. Activated astrocytes and increased cleaved caspase 3 levels confirmed an upregulated brain inflammation and apoptosis in aged cirrhotic mice. Although astrocyte activation has been described in experimental cirrhosis [60], the specific age-contributing role in increased number of GFAP-positive astrocytes has also been reported [61, 62], supporting results presented herein. Similarly, activation of caspase 3 has been involved in age-related diseases [63-65].

Aged cirrhosis showed a clearly skewed CD8^+^ T cell subset expansion along with a low functional activity in the brain. In response to astrocyte activation, cytotoxic activity is disseminated by NK and CD8^+^ T cells to produce neuroinflammation [66]. The fact that we observe a restrained cytotoxic activity in the brain of old cirrhotic mice together with the expansion of CD8^+^ T cells may suggest either a neuroprotective reprogrammed immunophenotype, as shown in acute brain injury during early stages of ischemic stroke [66], or rather an exhausted behavior more related to chronic damage, as that shown in chronic viral infection [67], for this population in cirrhosis during aging. This fact is supported by the correlation observed between expanded CD8^+^ T cell population in the brain with neuromotor and cognitive functions in our series of animals, where old cirrhotic mice perform worser than the rest of groups and, additionally, by the correlation between perforin brain levels and memory capacities of mice, where old cirrhotic mice exhibited a more severe dysfunction.

Some limitations of the present study must be acknowledged. First, the transversal design of the study does not allow to provide insight on the relationship between the progression of liver disease and neurocognitive decline. Future longitudinal studies may help better disentangle how the liver damage progress is influenced by aging. Secondly, although the CCl_4_-induced liver damage represents a well-established model of cirrhosis, the use of additional chronic liver disease models, as well as specifically exploring gender differences, would also provide valuable translational information on human liver disease of different etiologies. Finally, although our study was focused on the liver-brain aspects influenced by aging during cirrhosis, other systemic effects such as cardiometabolic or renal complications are surely also influenced and should be covered by future studies on aging in cirrhosis.

In summary, aging aggravates liver fibrosis, worsens neuromotor and cognitive functions and shifts liver and brain adaptive T cell profiles compromising the BBB integrity in experimental advanced chronic liver disease. Results strengthen the role of aging in the liver-brain axis and neuroinflammation in cirrhosis.

## Supplementary Materials

The Supplementary data can be found online at: www.aginganddisease.org/EN/10.14336/AD.2024.0932.

## References

[1] Pimpin L, Cortez-Pinto H, Negro F, Corbould E, Lazarus JV, Webber L, et al. (2018). Burden of liver disease in Europe: Epidemiology and analysis of risk factors to identify prevention policies. J Hepatol, 69:718-735. 10.1016/j.jhep.2018.05.011 29777749

[2] Moon AM, Singal AG, Tapper EB (2020). Contemporary Epidemiology of Chronic Liver Disease and Cirrhosis. Clin Gastroenterol Hepatol, 18:2650-2666. 10.1016/j.cgh.2019.07.060 PMC7007353 31401364

[3] Bajaj JS, Hafeezullah M, Hoffmann RG, Varma RR, Franco J, Binion DG, et al. (2008). Navigation skill impairment: Another dimension of the driving difficulties in minimal hepatic encephalopathy. Hepatology, 47:596-604. 10.1002/hep.22032 18000989

[4] Romero-Gomez M, Boza F, Garcia-Valdecasas MS, Garcia E, Aguilar-Reina J (2001). Subclinical hepatic encephalopathy predicts the development of overt hepatic encephalopathy. Am J Gastroenterol, 96:2718-2723. 10.1111/j.1572-0241.2001.04130.x 11569701

[5] Ezaz G, Murphy SL, Mellinger J, Tapper EB (2018). Increased Morbidity and Mortality Associated with Falls Among Patients with Cirrhosis. Am J Med, 131:645-650 e642. 10.1016/j.amjmed.2018.01.026 29453941

[6] Butterworth RF (2019). Hepatic Encephalopathy in Cirrhosis: Pathology and Pathophysiology. Drugs, 79:17-21. 10.1007/s40265-018-1017-0 PMC6416236 30706423

[7] Thomson AW, Knolle PA (2010). Antigen-presenting cell function in the tolerogenic liver environment. Nat Rev Immunol, 10:753-766. 10.1038/nri2858 20972472

[8] Shetty S, Lalor PF, Adams DH (2018). Liver sinusoidal endothelial cells - gatekeepers of hepatic immunity. Nat Rev Gastroenterol Hepatol, 15:555-567. 10.1038/s41575-018-0020-y PMC7096836 29844586

[9] Fouts DE, Torralba M, Nelson KE, Brenner DA, Schnabl B (2012). Bacterial translocation and changes in the intestinal microbiome in mouse models of liver disease. J Hepatol, 56:1283-1292. 10.1016/j.jhep.2012.01.019 PMC3357486 22326468

[10] Chen Y, Yang F, Lu H, Wang B, Lei D, Wang Y, et al. (2011). Characterization of fecal microbial communities in patients with liver cirrhosis. Hepatology, 54:562-572. 10.1002/hep.24423 21574172

[11] Albillos A, Hera Ad AL, Reyes E, Monserrat J, Munoz L, Nieto M, et al. (2004). Tumour necrosis factor-alpha expression by activated monocytes and altered T-cell homeostasis in ascitic alcoholic cirrhosis: amelioration with norfloxacin. [J] Hepatol., 40:624-631. 10.1016/j.jhep.2003.12.010 15030978

[12] Munoz L, Albillos A, Nieto M, Reyes E, Lledo L, Monserrat J, et al. (2005). Mesenteric Th1 polarization and monocyte TNF-alpha production: first steps to systemic inflammation in rats with cirrhosis. Hepatology, 42:411-419. 10.1002/hep.20799 16025514

[13] Lemmers A, Moreno C, Gustot T, Marechal R, Degre D, Demetter P, et al. (2009). The interleukin-17 pathway is involved in human alcoholic liver disease. Hepatology, 49:646-657. 10.1002/hep.22680 19177575

[14] Sun HQ, Zhang JY, Zhang H, Zou ZS, Wang FS, Jia JH (2012). Increased Th17 cells contribute to disease progression in patients with HBV-associated liver cirrhosis. J Viral Hepat, 19:396-403. 10.1111/j.1365-2893.2011.01561.x 22571901

[15] Albillos A, Lario M, Alvarez-Mon M (2014). Cirrhosis-associated immune dysfunction: distinctive features and clinical relevance. J Hepatol, 61:1385-1396. 10.1016/j.jhep.2014.08.010 25135860

[16] Cipollini V, Anrather J, Orzi F, Iadecola C (2019). Th17 and Cognitive Impairment: Possible Mechanisms of Action. Front Neuroanat, 13:95. 10.3389/fnana.2019.00095 PMC6877481 31803028

[17] Le Couteur DG, McLean AJ (1998). The aging liver. Drug clearance and an oxygen diffusion barrier hypothesis. Clin Pharmacokinet, 34:359-373. 10.2165/00003088-199834050-00003 9592620

[18] Angulo P, Keach JC, Batts KP, Lindor KD (1999). Independent predictors of liver fibrosis in patients with nonalcoholic steatohepatitis. Hepatology, 30:1356-1362. 10.1002/hep.510300604 10573511

[19] Hunt NJ, Kang SWS, Lockwood GP, Le Couteur DG, Cogger VC (2019). Hallmarks of Aging in the Liver. Comput Struct Biotechnol J, 17:1151-1161. 10.1016/j.csbj.2019.07.021 PMC6709368 31462971

[20] Maeso-Diaz R, Ortega-Ribera M, Lafoz E, Lozano JJ, Baiges A, Frances R, et al. (2019). Aging Influences Hepatic Microvascular Biology and Liver Fibrosis in Advanced Chronic Liver Disease. Aging Dis, 10:684-698. 10.14336/AD.2019.0127 PMC6675529 31440376

[21] Gomez-Hurtado I, Santacruz A, Peiro G, Zapater P, Gutierrez A, Perez-Mateo M, et al. (2011). Gut microbiota dysbiosis is associated with inflammation and bacterial translocation in mice with CCl4-induced fibrosis. PLoS.One., 6:e23037. 10.1371/journal.pone.0023037 PMC3146520 21829583

[22] Gracia-Sancho J, Lavina B, Rodriguez-Vilarrupla A, Garcia-Caldero H, Bosch J, Garcia-Pagan JC (2007). Enhanced vasoconstrictor prostanoid production by sinusoidal endothelial cells increases portal perfusion pressure in cirrhotic rat livers. J Hepatol, 47:220-227. 10.1016/j.jhep.2007.03.014 17459512

[23] Perez-Rial S, Garcia-Gutierrez MS, Molina JA, Perez-Nievas BG, Ledent C, Leiva C, et al. (2011). Increased vulnerability to 6-hydroxydopamine lesion and reduced development of dyskinesias in mice lacking CB1 cannabinoid receptors. Neurobiol Aging, 32:631-645. 10.1016/j.neurobiolaging.2009.03.017 19419794

[24] La Porta C, Bura SA, Llorente-Onaindia J, Pastor A, Navarrete F, Garcia-Gutierrez MS, et al. (2015). Role of the endocannabinoid system in the emotional manifestations of osteoarthritis pain. Pain, 156:2001-2012. 10.1097/j.pain.0000000000000260 PMC4770330 26067584

[25] Gines P, Krag A, Abraldes JG, Sola E, Fabrellas N, Kamath PS (2021). Liver cirrhosis. Lancet, 398:1359-1376. 10.1016/S0140-6736(21)01374-X 34543610

[26] Rose CF, Amodio P, Bajaj JS, Dhiman RK, Montagnese S, Taylor-Robinson SD, et al. (2020). Hepatic encephalopathy: Novel insights into classification, pathophysiology and therapy. J Hepatol, 73:1526-1547. 10.1016/j.jhep.2020.07.013 33097308

[27] Ramirez T, Li YM, Yin S, Xu MJ, Feng D, Zhou Z, et al. (2017). Aging aggravates alcoholic liver injury and fibrosis in mice by downregulating sirtuin 1 expression. J Hepatol, 66:601-609. 10.1016/j.jhep.2016.11.004 PMC5316497 27871879

[28] Consolo M, Amoroso A, Spandidos DA, Mazzarino MC (2009). Matrix metalloproteinases and their inhibitors as markers of inflammation and fibrosis in chronic liver disease (Review). Int J Mol Med, 24:143-152. 10.3892/ijmm_00000217 19578787

[29] Roderfeld M (2018). Matrix metalloproteinase functions in hepatic injury and fibrosis. Matrix Biol, 68-69:452-462. 10.1016/j.matbio.2017.11.011 29221811

[30] Collins BH, Holzknecht ZE, Lynn KA, Sempowski GD, Smith CC, Liu S, et al. (2013). Association of age-dependent liver injury and fibrosis with immune cell populations. Liver Int, 33:1175-1186. 10.1111/liv.12202 PMC4151465 23710620

[31] Kim IH, Xu J, Liu X, Koyama Y, Ma HY, Diggle K, et al. (2016). Aging increases the susceptibility of hepatic inflammation, liver fibrosis and aging in response to high-fat diet in mice. Age (Dordr), 38:291-302. 10.1007/s11357-016-9938-6 PMC5061686 27578257

[32] Albillos A, de Gottardi A, Rescigno M (2020). The gut-liver axis in liver disease: Pathophysiological basis for therapy. J Hepatol, 72:558-577. 10.1016/j.jhep.2019.10.003 31622696

[33] Fabre T, Kared H, Friedman SL, Shoukry NH (2014). IL-17A enhances the expression of profibrotic genes through upregulation of the TGF-beta receptor on hepatic stellate cells in a JNK-dependent manner. J Immunol, 193:3925-3933. 10.4049/jimmunol.1400861 PMC4185218 25210118

[34] Matsuda KM, Kotani H, Hisamoto T, Kuzumi A, Fukasawa T, Yoshizaki-Ogawa A, et al. (2024). Dual blockade of interleukin-17A and interleukin-17F as a therapeutic strategy for liver fibrosis: Investigating the potential effect and mechanism of brodalumab. Cytokine, 178:156587. 10.1016/j.cyto.2024.156587 38531177

[35] Meng F, Wang K, Aoyama T, Grivennikov SI, Paik Y, Scholten D, et al. (2012). Interleukin-17 signaling in inflammatory, Kupffer cells, and hepatic stellate cells exacerbates liver fibrosis in mice. Gastroenterology, 143:765-776 e763. 10.1053/j.gastro.2012.05.049 PMC3635475 22687286

[36] Chiaramonte MG, Donaldson DD, Cheever AW, Wynn TA (1999). An IL-13 inhibitor blocks the development of hepatic fibrosis during a T-helper type 2-dominated inflammatory response. J Clin Invest, 104:777-785. 10.1172/JCI7325 PMC408441 10491413

[37] Pellicoro A, Ramachandran P, Iredale JP, Fallowfield JA (2014). Liver fibrosis and repair: immune regulation of wound healing in a solid organ. Nat Rev Immunol, 14:181-194. 10.1038/nri3623 24566915

[38] Castellino F, Huang AY, Altan-Bonnet G, Stoll S, Scheinecker C, Germain RN (2006). Chemokines enhance immunity by guiding naive CD8+ T cells to sites of CD4+ T cell-dendritic cell interaction. Nature, 440:890-895. 10.1038/nature04651 16612374

[39] Ozga AJ, Chow MT, Lopes ME, Servis RL, Di Pilato M, Dehio P, et al. (2022). CXCL10 chemokine regulates heterogeneity of the CD8(+) T cell response and viral set point during chronic infection. Immunity, 55:82-97 e88. 10.1016/j.immuni.2021.11.002 PMC8755631 34847356

[40] Wiggins BG, Pallett LJ, Li X, Davies SP, Amin OE, Gill US, et al. (2022). The human liver microenvironment shapes the homing and function of CD4(+) T-cell populations. Gut, 71:1399-1411. 10.1136/gutjnl-2020-323771 PMC9185819 34548339

[41] Ozkazanc D, Yoyen-Ermis D, Tavukcuoglu E, Buyukasik Y, Esendagli G (2016). Functional exhaustion of CD4(+) T cells induced by co-stimulatory signals from myeloid leukaemia cells. Immunology, 149:460-471. 10.1111/imm.12665 PMC5095494 27565576

[42] Stelma F, de Niet A, Sinnige MJ, van Dort KA, van Gisbergen K, Verheij J, et al. (2017). Human intrahepatic CD69 + CD8+ T cells have a tissue resident memory T cell phenotype with reduced cytolytic capacity. Sci Rep, 7:6172. 10.1038/s41598-017-06352-3 PMC5522381 28733665

[43] Pfister D, Nunez NG, Pinyol R, Govaere O, Pinter M, Szydlowska M, et al. (2021). NASH limits anti-tumour surveillance in immunotherapy-treated HCC. Nature, 592:450-456. 10.1038/s41586-021-03362-0 PMC8046670 33762733

[44] Sim BC, Kang YE, You SK, Lee SE, Nga HT, Lee HY, et al. (2023). Hepatic T-cell senescence and exhaustion are implicated in the progression of fatty liver disease in patients with type 2 diabetes and mouse model with nonalcoholic steatohepatitis. Cell Death Dis, 14:618. 10.1038/s41419-023-06146-8 PMC10514041 37735474

[45] Lebosse F, Gudd C, Tunc E, Singanayagam A, Nathwani R, Triantafyllou E, et al. (2019). CD8(+)T cells from patients with cirrhosis display a phenotype that may contribute to cirrhosis-associated immune dysfunction. EBioMedicine, 49:258-268. 10.1016/j.ebiom.2019.10.011 PMC6945243 31678004

[46] Jaffe A, Lim JK, Jakab SS (2020). Pathophysiology of Hepatic Encephalopathy. Clin Liver Dis, 24:175-188. 10.1016/j.cld.2020.01.002 32245525

[47] Ampuero J, Simon M, Montoliu C, Jover R, Serra MA, Cordoba J, et al. (2015). Minimal Hepatic Encephalopathy and Critical Flicker Frequency Are Associated With Survival of Patients With Cirrhosis. Gastroenterology, 149:1483-1489. 10.1053/j.gastro.2015.07.067 26299413

[48] Weiss N, Rosselli M, Mouri S, Galanaud D, Puybasset L, Agarwal B, et al. (2017). Modification in CSF specific gravity in acutely decompensated cirrhosis and acute on chronic liver failure independent of encephalopathy, evidences for an early blood-CSF barrier dysfunction in cirrhosis. Metab Brain Dis, 32:369-376. 10.1007/s11011-016-9916-9 27730496

[49] Roman E, Cordoba J, Torrens M, Guarner C, Soriano G (2013). Falls and cognitive dysfunction impair health-related quality of life in patients with cirrhosis. Eur J Gastroenterol Hepatol, 25:77-84. 10.1097/MEG.0b013e3283589f49 22954704

[50] Soriano G, Roman E, Cordoba J, Torrens M, Poca M, Torras X, et al. (2012). Cognitive dysfunction in cirrhosis is associated with falls: a prospective study. Hepatology, 55:1922-1930. 10.1002/hep.25554 22213000

[51] Thomsen KL, Sorensen M, Kjaergaard K, Eriksen PL, Lauridsen MM, Vilstrup H (2024). Cerebral Aspects of Portal Hypertension: Hepatic Encephalopathy. Clin Liver Dis, 28:541-554. 10.1016/j.cld.2024.03.008 38945642

[52] Llansola M, Ahabrach H, Errami M, Cabrera-Pastor A, Addaoudi K, Felipo V (2013). Impaired release of corticosterone from adrenals contributes to impairment of circadian rhythms of activity in hyperammonemic rats. Arch Biochem Biophys, 536:164-170. 10.1016/j.abb.2013.01.009 23376587

[53] Arenas YM, Martinez-Garcia M, Llansola M, Felipo V (2022). Enhanced BDNF and TrkB Activation Enhance GABA Neurotransmission in Cerebellum in Hyperammonemia. Int J Mol Sci, 23. 10.3390/ijms231911770 PMC9570361 36233065

[54] Franca MER, Ramos R, Oliveira WH, Duarte-Silva E, Araujo SMR, Los DB, et al. (2019). Tadalafil restores long-term memory and synaptic plasticity in mice with hepatic encephalopathy. Toxicol Appl Pharmacol, 379:114673. 10.1016/j.taap.2019.114673 31323263

[55] Cauli O, Lopez-Larrubia P, Rodrigo R, Agusti A, Boix J, Nieto-Charques L, et al. (2011). Brain region-selective mechanisms contribute to the progression of cerebral alterations in acute liver failure in rats. Gastroenterology, 140:638-645. 10.1053/j.gastro.2010.10.043 20977905

[56] Ott P, Larsen FS (2004). Blood-brain barrier permeability to ammonia in liver failure: a critical reappraisal. Neurochem Int, 44:185-198. 10.1016/s0197-0186(03)00153-0 14602081

[57] Munoz L, Caparros E, Albillos A, Frances R (2023). The shaping of gut immunity in cirrhosis. Front Immunol, 14:1139554. 10.3389/fimmu.2023.1139554 PMC10141304 37122743

[58] Dhanda S, Sandhir R (2018). Blood-Brain Barrier Permeability Is Exacerbated in Experimental Model of Hepatic Encephalopathy via MMP-9 Activation and Downregulation of Tight Junction Proteins. Mol Neurobiol, 55:3642-3659. 10.1007/s12035-017-0521-7 28523565

[59] Liu Y, Zhang M, Hao W, Mihaljevic I, Liu X, Xie K, et al. (2013). Matrix metalloproteinase-12 contributes to neuroinflammation in the aged brain. Neurobiol Aging, 34:1231-1239. 10.1016/j.neurobiolaging.2012.10.015 23159549

[60] Wright GA, Sharifi Y, Newman TA, Davies N, Vairappan B, Perry HV, et al. (2014). Characterisation of temporal microglia and astrocyte immune responses in bile duct-ligated rat models of cirrhosis. Liver Int, 34:1184-1191. 10.1111/liv.12481 24528887

[61] Nichols NR, Day JR, Laping NJ, Johnson SA, Finch CE (1993). GFAP mRNA increases with age in rat and human brain. Neurobiol Aging, 14:421-429. 10.1016/0197-4580(93)90100-p 8247224

[62] David JP, Ghozali F, Fallet-Bianco C, Wattez A, Delaine S, Boniface B, et al. (1997). Glial reaction in the hippocampal formation is highly correlated with aging in human brain. Neurosci Lett, 235:53-56. 10.1016/s0304-3940(97)00708-8 9389594

[63] Rissman RA, Poon WW, Blurton-Jones M, Oddo S, Torp R, Vitek MP, et al. (2004). Caspase-cleavage of tau is an early event in Alzheimer disease tangle pathology. J Clin Invest, 114:121-130. 10.1172/JCI20640 PMC437967 15232619

[64] Su JH, Zhao M, Anderson AJ, Srinivasan A, Cotman CW (2001). Activated caspase-3 expression in Alzheimer's and aged control brain: correlation with Alzheimer pathology. Brain Res, 898:350-357. 10.1016/s0006-8993(01)02018-2 11306022

[65] Su JH, Nichol KE, Sitch T, Sheu P, Chubb C, Miller BL, et al. (2000). DNA damage and activated caspase-3 expression in neurons and astrocytes: evidence for apoptosis in frontotemporal dementia. Exp Neurol, 163:9-19. 10.1006/exnr.2000.7340 10785439

[66] Zhang Z, Duan Z, Cui Y (2023). CD8(+) T cells in brain injury and neurodegeneration. Front Cell Neurosci, 17:1281763. 10.3389/fncel.2023.1281763 PMC10702747 38077952

[67] Wherry EJ, Blattman JN, Murali-Krishna K, van der Most R, Ahmed R (2003). Viral persistence alters CD8 T-cell immunodominance and tissue distribution and results in distinct stages of functional impairment. J Virol, 77:4911-4927. 10.1128/JVI.77.8.4911-4927.2003 PMC152117 12663797

