# Supplementary material for: Aging Deteriorates Blood Brain Barrier Function and Polarizes Adaptive T Cell Expansion Contributing to Neurocognitive Damage in Experimental Cirrhosis
Source: Aging Dis. 2024 Oct 12;16(5):3112–27. doi: 10.14336/AD.2024.0932 (PMC12339172; doi:10.14336/AD.2024.0932)
Supplement: Supplementary file 1 — The Supplementary data can be found online at: www.aginganddisease.org/EN/10.14336/AD.2024.0932. [file AD-16-5-3112-s.pdf]

SUPPLEMENTARY DATA

Aging Deteriorates Blood Brain Barrier Function and Polarizes Adaptive T Cell Expansion Contributing to Neurocognitive Damage in Experimental Cirrhosis

Sebastián Martínez-López, María Salud García-Gutiérrez, Francisco Navarrete, Isabel Gómez-Hurtado, Pedro Zapater, Enrique Ángel, Oriol Juanola, Juan L López-Cánovas, Paula Boix, Manel C Hadid, Amaya Puig-Kröger, Manuel D Gahete, Jorge Manzanares, Esther Caparrós, Rubén Francés

© 2024. Martínez-López **S.** et al. Published online at http://www.aginganddisease.org/EN/10.14336/AD.2024.0932

SUPPLEMENTARY DATA

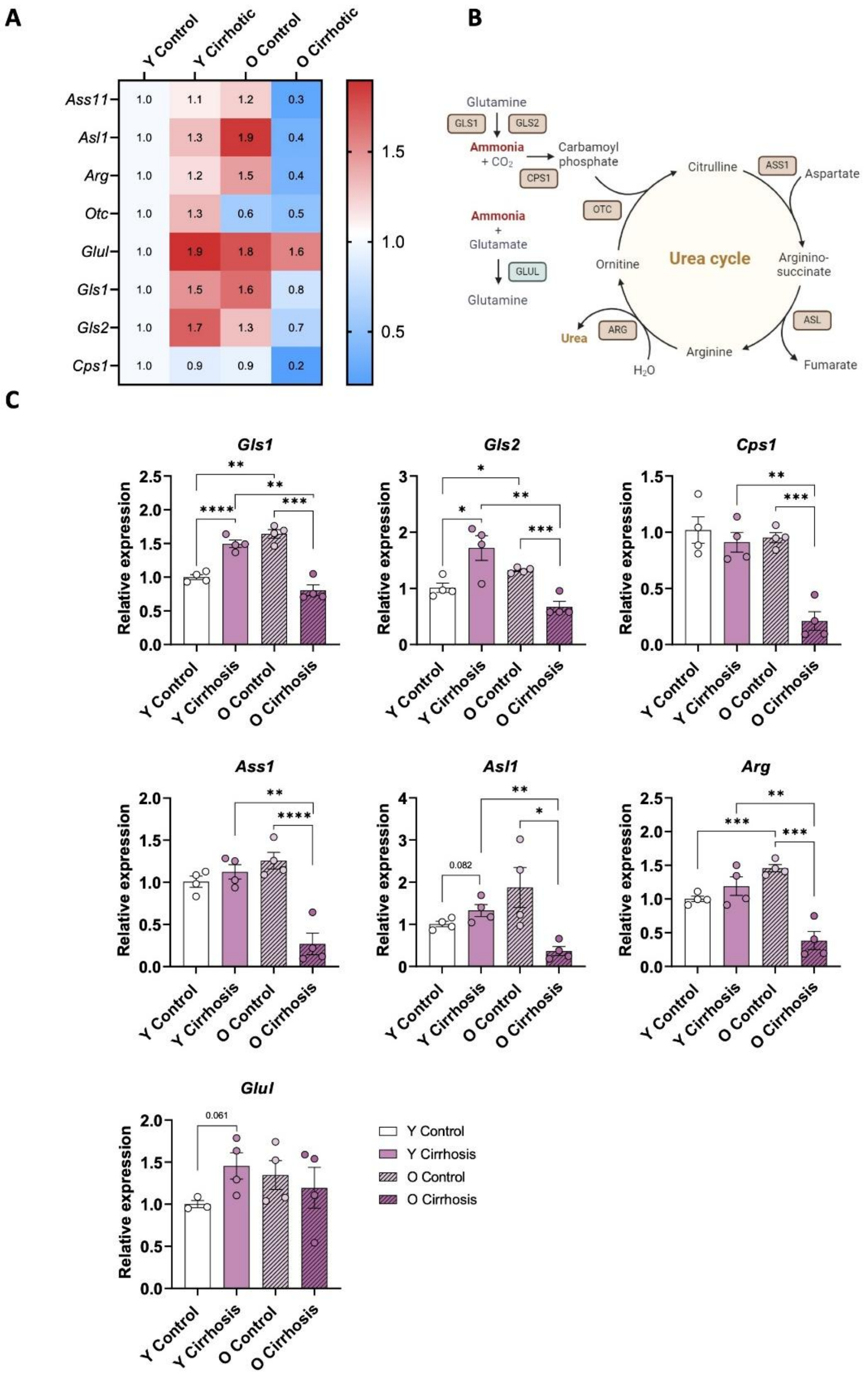

© 2024. Martínez-López **S.** et al. Published online at http://www.aginganddisease.org/EN/10.14336/AD.2024.0932

SUPPLEMENTARY DATA

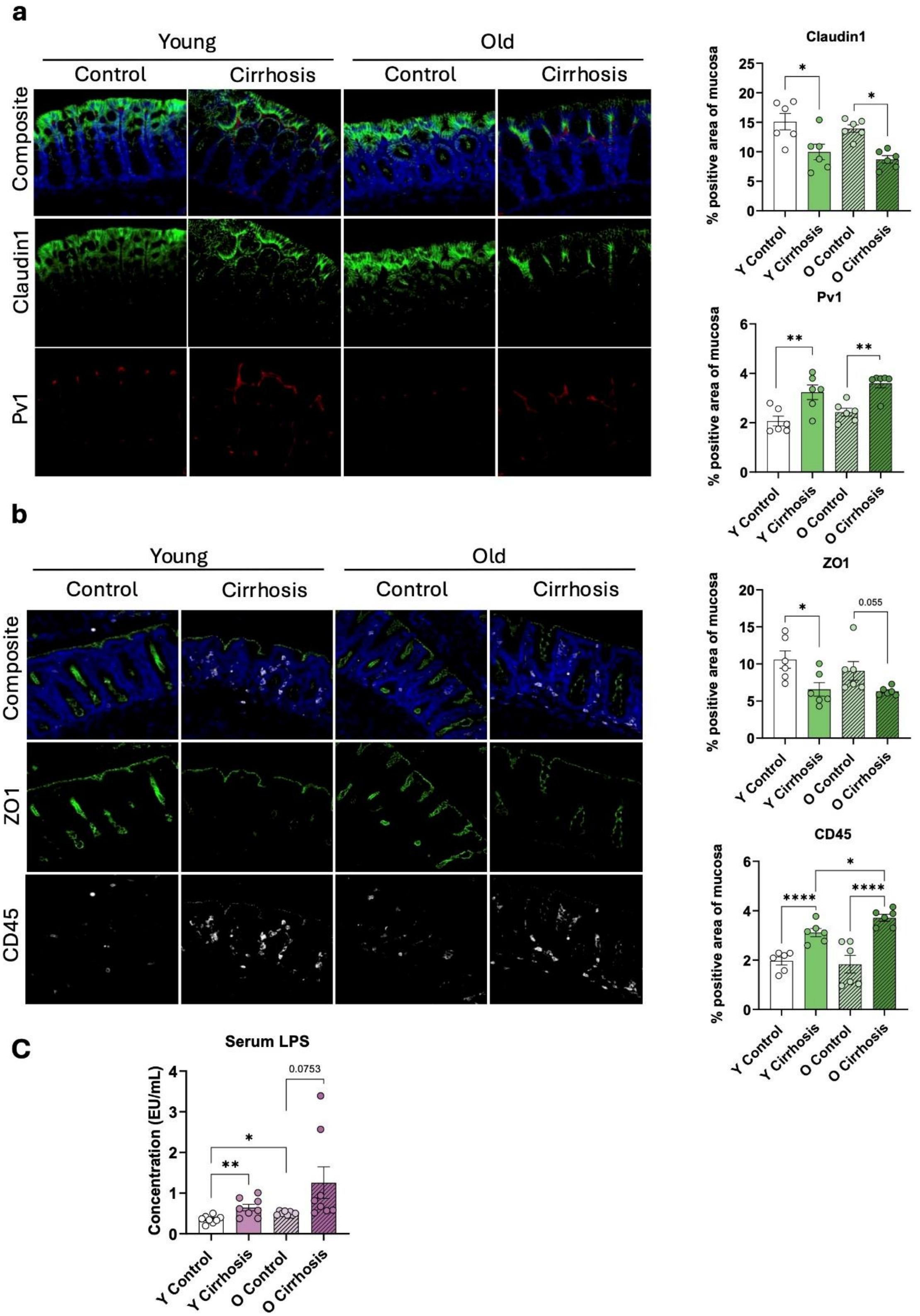

© 2024. Martínez-López **S.** et al. Published online at http://www.aginganddisease.org/EN/10.14336/AD.2024.0932

SUPPLEMENTARY DATA

Supplementary Figure 2. Gut barrier integrity in young and old mice with cirrhosis. Representative Immunofluorescence of proteins involved in the integrity of the gut barrier in sections of paraffin-embedded mouse colon tissue. (A) Expression of Claudin1 and Pv1 (B) Expression of Zo1 and CD45. Expression was blindly measured by densitometry in user-specified regions of interest (ROIs) as percentage of positive area of mucosa. Area of mucosa was quantified using the DAPI channel using the ImageJ software. Mean ± standard deviation is represented. (C) Quantification of endotoxin in serum as an indicative of gut permeability (8 animals/group). Abbreviations: Plasmalemma vesicle-associated protein (Pv1), Zonula Occludens1 (Zo1), Cluster of differentiation (CD), Lipopolysaccharide/Endotoxin (LPS), Young (Y) and Old (O). P values are indicated as follows (*) p < 0.05; (**) p < 0.01; (***) p < 0.001; and (****) p < 0.0001.

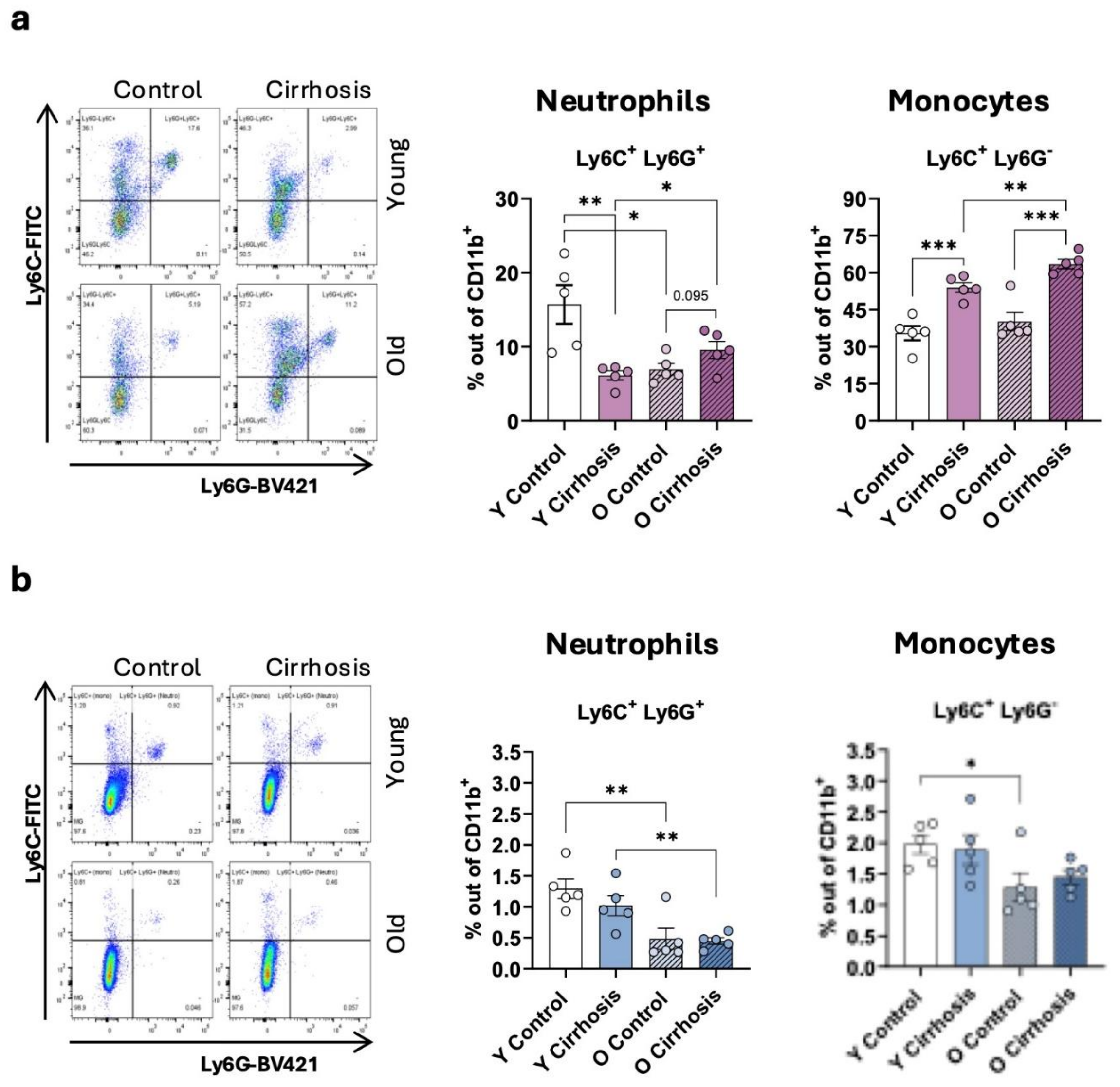

Supplementary Figure 3. Innate immune system alterations in brains and livers from young and old mice with cirrhosis. Representative dot plot images from flow cytometry analysis of $CD11b^+$ cells of the liver (A) and the brain (B) expressing Ly6C alone for monocytes or in combination with Ly6G for neutrophils. Data is expressed as percentage of positive cells of the previous gate. Mean ± standard deviation is represented (5 animals/group). Abbreviations: Cluster of differentiation (CD), Lymphocyte antigen 6 (Ly6), Young (Y) and Old (O). P values are indicated as follows (*) p < 0.05; (**) p < 0.01; (***) p < 0.001; and (****) p < 0.0001.

© 2024. Martínez-López **S.** et al. Published online at http://www.aginganddisease.org/EN/10.14336/AD.2024.0932

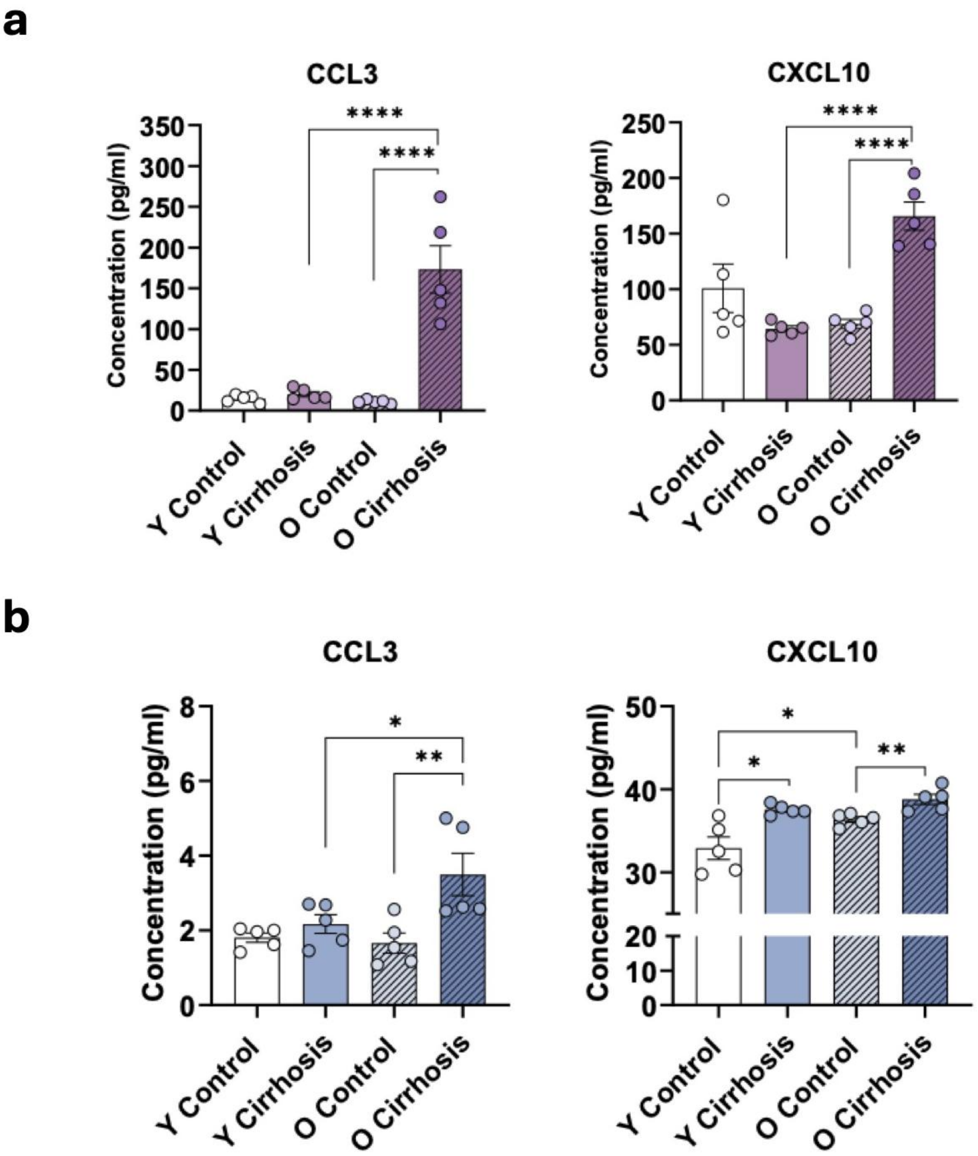

Supplementary Figure 4. Chemokines involved in CD8$^+$ cells recruitment in brains and livers from young and old mice with cirrhosis. CCL3 and CXCL10 levels in livers (A) and brain lysates (B) measured by a multi-analyte flow assay kit. Mean ± standard deviation is represented (5 animals/group). Abbreviations: C-C Motif Chemokine Ligand 3 (CCL3), C-X-C Motif Chemokine Ligand 10 (CXCL10), Young (Y) and Old (O). P values are indicated as follows (*) $p < 0.05$; (**) $p < 0.01$; (***) $p < 0.001$; and (****) $p < 0.0001$.

© 2024. Martínez-López **S.** et al. Published online at http://www.aginganddisease.org/EN/10.14336/AD.2024.0932

SUPPLEMENTARY DATA

Supplementary Table 1. Antibodies for flow cytometry.

PROTEIN	TARGET	DILUTION	DISTRIBUTOR	CONJUGATION
**CD3**	Mouse	1:200	Biolegend	PE
**CD4**	Mouse	1:200	Biolegend	FITC
**CD8**	Mouse	1:100	Biolegend	BV510
**LY6C**	Mouse	1:200	Biolegend	FITC
**LY6G**	Mouse	1:200	Biolegend	BV521
**CD11B**	Mouse	1:200	Biolegend	PE-Cy7
**CD69**	Mouse	1:100	Biolegend	APC
**PD-1**	Mouse	1:100	Biolegend	PE
**IL-17**	Mouse	1:50	Biolegend	APC
**IL10**	Mouse	1:50	Biolegend	PE-Cy7
**IL-4**	Mouse	1:50	Biolegend	BV421

Supplementary Table 2. Antibodies for histological analysis.

PROTEIN	TARGET	HOST	DILUTION	REFERENCE	TECHNIQUE
**ASMA**	Mouse	Rabbit	1:200	D4K9N	IHC
**VIM**	Mouse	Rabbit	1:100	D21H3	IHC
**COL1A1**	Mouse	Rabbit	1:300	AB765P	IHC
**CD45**	Mouse	Goat	1:300	AF114	IHC
**CD3**	Mouse	Rabbit	1:100	D7A6E	IHC
**GFAP**	Mouse	Goat	1:500	13-0300	IF
**ZO1**	Mouse	Rabbit	1:300	PA5-28858	IF
**CLAUDIN1**	Mouse	Rabbit	1:300	ab15098	IF
**CD45**	Mouse	Goat	1:200	AF114	IF
**PV1**	Mouse	Rat	1:200	550563	IF
**CONJUGATION**	TARGET	HOST	DILUTION	REFERENCE	TECHNIQUE
**A488**	Anti-goat	Donkey	1:300	ab150133	IF
**A488**	Anti-mouse	Donkey	1:300	406416	IF
**A594**	Anti-rat	Donkey	1:300	ab150156	IF
**A647**	Anti-goat	Donkey	1:300	A-21447	IF
**BIOTIN**	Anti-rabbit	Horse	1:250	BA-1100	IHC
**BIOTIN**	Anti-goat	Horse	1:250	BA-9200	IHC

© 2024. Martínez-López **S.** et al. Published online at http://www.aginganddisease.org/EN/10.14336/AD.2024.0932

SUPPLEMENTARY DATA

Supplementary Table 3. Neurological severity score (NSS)

TASKS	POINTS
**INABILITY TO WALK ON A BEAM**	
**3 CM WIDE**	1
**2 CM WIDE**	1
**1 CM WIDE**	1
**INABILITY TO BALANCE ON A BEAM 0.5 CM WIDE FOR**	
**20 S**	1
**40 S**	1
**60 S**	1
**INABILITY TO BALANCE ON A ROUND STICK 0.5 CM IN DIAMETER FOR**	
**10S**	1
**INABILITY TO WALK STRAIGHT**	1
**INABILITY TO EXIT FROM A CIRCLE 30 CM IN DIAMETER WHEN LEFT IN ITS CENTER WITHIN**	
**20 S**	1
**40 S**	1
**60 S**	1
**TOTAL SCORE**	**11**

Supplementary Table 4. Primers for qPCR.

GENE	TARGET	FORWARD PRIMER	REVERSE PRIMER
**COL1A1**	Mouse	actcgaacgggaatccat	gtgttccctactcagccg
**TIMP1**	Mouse	ccagaaccgcagtgaagagt	gaaacactgtgcacacccca
**ACTA2**	Mouse	gtcccagacatcagggagtaa	tcggatacttcagcgtcagga
**MMP2**	Mouse	cttgttgcccaggaaagtgaa	ccgaggactatgaccgggata
**ARG**	Mouse	tgtgaagaacccacggtctg	gcaccacactgactcttcca
**ASL1**	Mouse	aagtggagccctgaagaaacc	aaatcccccagcccactcat
**ASS1**	Mouse	tccagtgcactctacgagga	caatctccacctgtctgcga
**CPS1**	Mouse	cagcctacagcctcaactg	tgtccaatttgtttgtaaccagtgt
**GLUL**	Mouse	gcaatggaattctcgtgccg	gcagccactgctttttccaa
**GLS1**	Mouse	tgcgaacatctgatcccagg	agcatgacaccatctgacgtt
**GLS2**	Mouse	atcttagccaggacacgctg	aggggagaaagagaacgact
**ACTB**	Mouse	atggctcgctcggtgaccct	ttctccggtgggtggcgtga
**MMP9**	Mouse	gctgactacgataaggacggca	tagtggtgcaggcagagtagga
**MMP12**	Mouse	cacacttcccaggaatcaagcc	tttggtgacacgacggaacagg

© 2024. Martínez-López **S.** et al. Published online at http://www.aginganddisease.org/EN/10.14336/AD.2024.0932

SUPPLEMENTARY DATA

Supplementary Table 5. TaqMan probes for brain regions.

GENE	TARGET	BRAIN AREA	REFERENCE
**CRH**	Mouse	PVN	Mm01293920_s1
**NR3C1**	Mouse	HIP	Mm00433832_m1
**BDNF**	Mouse	HIP	Mm04230607_s1
**NTRK2**	Mouse	HIP	Mm00435422_m1
**18S**	Mouse	Reference gene	Mm03928990_g1

Supplementary Table 6. Antibodies for Western blot.

PROTEIN	TARGET	HOST	DILUTION	REFERENCE	TECHNIQUE
**ZO1**	Mouse	Rabbit	1:1000	PA5-28858	WB
**CLAUDIN1**	Mouse	Rabbit	1:1000	ab15098	WB
**CASP3-CLEAVED**	Mouse	Rabbit	1:2000	9661	WB
**GRANZYME B**	Mouse	Rabbit	1:750	4275	WB
**PERFORIN**	Mouse	Rabbit	1:750	3693	WB
**B-ACTIN**	Mouse	Mouse	1:10000	A5441	WB
**CONJUGATION**	Target	Host	Dilution	Reference	Technique
**HRP**	Anti-rabbit	Goat	1:3000	31460	WB
**HRP**	Anti-mouse	Goat	1:5000	31430	WB

© 2024. Martínez-López **S.** et al. Published online at http://www.aginganddisease.org/EN/10.14336/AD.2024.0932
